# Cannabis Constituents and Acetylcholinesterase Interaction: Molecular Docking, In Vitro Studies and Association with *CNR1 rs806368* and *ACHE rs17228602*

**DOI:** 10.3390/biom10050758

**Published:** 2020-05-13

**Authors:** Tiyyaba Furqan, Sidra Batool, Rabia Habib, Mamoona Shah, Huba Kalasz, Ferenc Darvas, Kamil Kuca, Eugenie Nepovimova, Sajida Batool, Syed M Nurulain

**Affiliations:** 1 Department of Biosciences, COMSATS University Islamabad, Islamabad 45550, Pakistan; tiyyaba.furqan@outlook.com (T.F.); rabiahabib@comsats.edu.pk (R.H.); maimoona.shah@outlook.com (M.S.); sajida.batool@comsats.edu.pk (S.B.); 2 Department of Biosciences, Bioinformatics laboratory, COMSATS University Islamabad, Islamabad 45550, Pakistan; sidra.batool@comsats.edu.pk; 3 Department of Pharmacology and Pharmacotherapy, Semmelweis University, 1089 Budapest, Hungary; drkalasz@gmail.com; 4 ComInnex Inc., 1031 Budapest, Hungary; ferenc.darvas@cominnex.com; 5 Department of Chemistry, Faculty of Science, University of Hradec Kralove, 50003 Hradec Kralove, Czech Republic; eugenie.nepovimova@uhk.cz

**Keywords:** acetylcholinesterase, cannabis, cholinergic, rs806368, rs17228602

## Abstract

The study documented here was aimed to find the molecular interactions of some of the cannabinoid constituents of cannabis with acetylcholinesterase (AChE). Molecular docking and LogP determination were performed to predict the AChE inhibitory effect and lipophilicity. AChE enzyme activity was measured in the blood of cannabis addicted human subjects. Further, genetic predisposition to cannabis addiction was investigated by association analysis of cannabinoid receptor 1 (*CNR1*) single nucleotide polymorphism (SNP) rs806368 and *ACHE* rs17228602 using restriction fragment length polymorphism (RFLP) method. All the understudied cannabis constituents showed promising binding affinities with AChE and are lipophilic in nature. The AChE activity was observed to be indifferent in cannabis addicted and non-addicted healthy controls. There was no significant association with *CNR1* SNP rs806368 and *ACHE* rs17228602. The study concludes that in silico prediction for individual biomolecules of cannabis is different from in vivo physiological action in human subjects when all are present together. However, for a deeper mechanistic insight into these interactions and association, multi-population studies are suggested. Further studies to explore the inhibitory potential of different cannabis constituents for intended AChE inhibitor-based drug are warranted.

## 1. Introduction

Cannabis is commonly referred as marihuana, marijuana, hashish and hash and is obtained from plant *Cannabis sativa* L. The medicinal use of cannabis has been documented from the Middle East and Asia since sixth century B.C. [1]. However, presently, it is one of the leading drugs of substance-abuse globally. Its use or possession comes under the criminal act in most of the countries, though legalized for medical and recreational use in some states of USA and European countries. More than five hundred biologically active molecules have been identified [2] from cannabis and are, categorized as cannabinoids and non-cannabinoids. Tetrahydrocannabinol (THC) is considered the most abundant constituent present in cannabis. The true interest for THC in cannabis is due to its psychotropic potential. The compounds in psychotropic preparation of cannabis are target of receptors present in endocannabinoid system (ECS) [3]. It is suggested that the risk of mental disorders, drug abuse and dependence increases by use of cannabis and other illicit drugs [4]. Research on the medicinal use of cannabis and its harmful effect on brain function is ongoing [5,6]. In the recent past, use of constituents of cannabis particularly, THC—a psychoactive and cannabidiol—a non-psychoactive cannabinoid [7] for Alzheimer treatment has got more attention [8,9,10]. Watt and Kari (2017) found that cannabidiol masks the psychoactive properties of THC when both are present together. The cannabis use reduces the feelings of anxiety diminishing stress and increasing relaxation [11].

Acetylcholine (ACh), a neurotransmitter of the cholinergic system is found to play a significant role in the treatment of numerous psychiatric disorders [12]. Acetylcholinesterase enzyme (AChE) switches off the transmission of neural impulse by hydrolyzing the acetylcholine rapidly in cholinergic pathway in peripheral and central nervous system. Therefore, inhibitors of acetylcholinesterase have been used historically for the treatment of various neuropathological conditions [13]. On the other hand, Terranova and coworkers [14] and many others reported the involvement of cannabinoids in memory and cognitive impairment. Cannabinoid like THC analogues; (−)-delta 8-THC-dimethyl-heptyl (DMH), (−)-delta 9-THC and (−)- delta 8-THC decreases the production of acetylcholine in a dose-dependent way in the hippocampus [15], while activation of muscarinic cholinergic receptors in this area increase the release of endocannabinoid (ECB) [16]. Studies in rats showed increased AChE activity in brain upon treatment with cannabis extract (rich in delta ^9^-THC). The increase in AChE activity could result in decrease of ACh in the brain [15]. An earlier study though has found no difference in AChE levels in hashish smokers [17]. However, they speculated the indirect effect of THC on AChE. Increased AChE in brain of rats were found when treated with cannabis resin [18]. Though no significant direct effect of THC on AChE was found by another study rather might have indirect effect on the cholinergic system through enzyme inhibitors like physostigmine [19]. More recently the promising potential of cannabidiol for Alzheimer treatment was reported [8]. Further work by Watt and Karl (2018) and Kim et al. (2019) found that combined use of cannabidiol and THC is rather a good candidate drug for treatment of Alzheimer [9,20]. However, clinical effectiveness of this combination still needs to be elaborated.

There have not been many studies investigating the role of AChE gene and single nucleotide polymorphism (SNPs) for a molecular and genetics insight into mechanisms and possible predisposition towards addiction and substance abuse. It has been previously reported that polymorphisms in cannabinoid receptor 1 gene, referred to as cannabinoid receptor 1 (*CNR1*)*,* are associated with tendency to substance abuse [21] and diseases like Alzheimer [22], psychotic disorders [23], alcohol dependence [22], and nicotine dependence [24]. Similarly, *ACHE* rs17228602 has been found to be associated with drug abuse vulnerability [25]. The aim of the present study was to investigate the interactions of some of the constituents of cannabis with AChE by molecular docking, in silico logP determination and measure the AChE in the blood of cannabis addicted individuals compared to healthy controls. Additionally, tentative association of *CNR1* (rs806368) and *ACHE* (rs17228602) SNPs with cannabis addiction was also investigated. The findings of this work will provide a ground to understand the interactions of cannabis and cholinergic components in human subjects and opening the possibilities for use of AChE inhibitor-based drugs. Furthermore, characterization of SNPs will uncover the putative genetic association with cannabis vulnerability and addition.

## 2. Materials and Methods

### 2.1. Molecular Docking

The molecular docking was performed to simulate the interactions between different cannabinoid (ligands) constituents of cannabis and AChE (protein) binding sites. First, the ligand orientation, position and conformation within the sites of protein were predicted; and then, analysis of binding affinity was carried out. The docking was performed according to Waqar and Sidra [26].

For docking analysis, 3D structure of AChE enzyme (PDB accession codes:4PQE) [27] was downloaded from RCSB Protein Data Bank (PDB) (http://www.rcsb.org). Three pdb entries belonging to human, mouse and electric ray were identified. Chimera [28] was used for alignment and superimposing the pdb structure of AChE of these three species; and the conserved residues were scanned. The PRALINE multiple sequence alignment toolbox was used to align the sequences of AChE from these three species. The binding sites residues are crucial in the ligand-receptor binding hence, utilized for structure-based drug designing [29]. The binding site residues for human AChE are given in Table 1. 3D structures of all the ligands i.e., tetrahydrocannabinol, cannabielsoin, cannabicyclol, cannabidiol, cannabigerol, cannabinol, cannabitriol, cannabivarin, paraoxon and donepezil were obtained using PubChem and Chemspider [30,31]. Molecular docking between residues of binding site of the receptor and ligands was performed by Autodock Vina PyRx version 0.8 [32] for Windows (available free at http://pyrx.sourceforge.net). Parameters used in Vina Search space for docking are given in Table 2. Briefly, first PyRx of the receptor was loaded into the program and then ligand file was loaded to perform molecular docking. The grid-size was established such that all possible binding site interactions pertaining to each ligand would be covered. Furthermore, the examination extended to ensure each ligand was in fact at its appropriate location with reference to the structure of the receptor. A total of nine runs were performed for each docking. The docking results were analyzed by comparing the binding interactions and binding energies between ligand and AChE receptor. Various ligand-receptor interactions like hydrogen bond, π-π interactions and Van der Waals forces were calculated.

### 2.2. LogP Determination

All logP values were calculated using Pallas 3812 of Prolog P (ComInnex, Budapest, Hungary). Pallas 3812 is a computer-assisted tool to be applied in drug research. It is an advanced version of MetabolExpert [33] that is used to calculate logP, metabolic pathway and several other characteristics of drugs and drug candidates.

### 2.3. Acetylcholinesterase Activity in Human Blood

#### 2.3.1. Sampling of Study Subjects

Cannabis addicted subjects were enlisted from different rehabilitation centers (New Roshni Center, Wada Rehab Center, Psychaid Hospital) in Islamabad, Pakistan. The study was approved by ethics review board (ERB) of the Department of Biosciences, COMSATS University Islamabad (CIIT/Bio/ERB/19/98). The study conformed to tenets of 1964 Declaration of Helsinki and its later amendments. After filling the consent form of patient, venous blood was taken in EDTA-vacutainer tubes (Atlas–Labovac Italiano, FL Medical, Torreglia PD, Italy). Data about age and gender were obtained. Forty-nine confirmed cannabis addicted individuals with average age (Mean ± SD, 29 ± 9) were included in this study. Apart from inclusion criteria, the fulfilment of exclusion criteria too was ensured which included any viral diseases, chronic diseases like diabetes, use of combination of drugs and drug abuse for less than three months. Age-matched forty-five non-addicted individuals were enlisted as controls. Only non-hemolysed blood were used for AChE measurements.

#### 2.3.2. Biochemical Measurement of AChE

AChE activity was measured using Ellamn’s method modified by Worek et al. [34]. This measurement was done in the presence of ethopropazine at 37 °C using 3 mL polystyrol cuvettes (Thomas Scientific, 1218871). Then, 1 mL blood dilutions, 2 mL 0.1M phosphate buffer pH 7.4, 100 μL DTNB (10 mM), 10 μL ethopropazine were mixed and incubated for 20 min at 37 °C. Then in the reaction mixture, 50 μL of acetylthiocholine (28.3 mmol/L) was added. Absorbance was measured by spectrophotometer (Specord 50 plus Number; 233H1280C, Analytic Jena, Germany) every minute for 5 min at 436 nm.

### 2.4. Primer Designing and Chemicals

Primer 3 version 0.4.0 software (http://bioinfo.ut.ee/primer3-0.4.0/primer3/) was used for designing of forward and reverse primer. NCBI Blast software (https://www.ncbi.nlm.nih.gov/tools/primer-blast/) was used to check the specificity of primer. Further testing was done by using In Silico PCR Tool (https://genome.ucsc.edu/cgi-bin/hgPcr). Primers were prepared by Macrogen (Rockville, MD, USA). Sequences of primers for both SNPs rs806368 and rs17228602 with their Tm and product sizes are given in Table 3. Chemicals for DNA extraction, PCR, RFLP analysis, Gel electrophoresis and AChE estimation were obtained from Thermo Fisher Scientific (Waltham, MA USA) and Sigma-Aldrich (St. Louis, MO, USA).

### 2.5. Genomic DNA Extraction and SNP Genotyping

**Figure 1:**
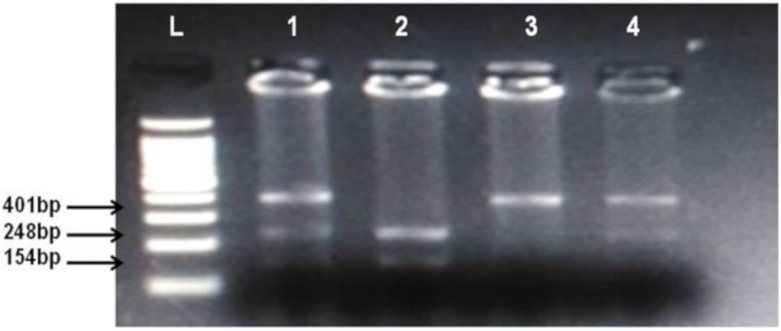
Identification of cannabinoid receptor 1 (CNR1) rs806368 polymorphism. Lanes L, DNA ladder, 1 and 4 show CT heterozygotes (Cleaved PCR product 248 bp, 154 bp and uncleaved PCR product 401 bp), 2 CC homozygote (cleaved PCR product into 248 bp and 154 bp fragments), 3 TT homozygote (uncleaved PCR product 401 bp).

**Figure 2:**
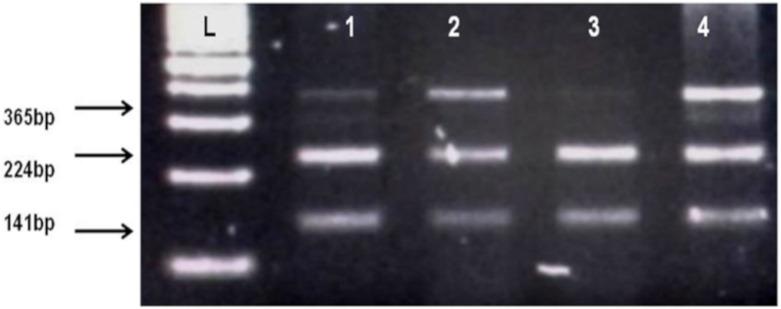
Identification of AChE rs17228602 polymorphism. L, DNA ladder, 1, 2, 4 show TC heterozygotes (Cleaved fragments 224 bp, 141 bp and uncleaved PCR products 365 bp), 3 shows CC homozygote (Cleaved PCR product into 224 bp and 141 bp fragments).

By using salting out method [35], genomic DNA was extracted from whole blood samples. Genotyping of *CNR1* (rs806368) and *ACHE* (rs17228602) was carried out by polymerase chain reaction and restriction fragment length polymorphism (PCR-RFLP) method. Forward and Reverse primer used in this method is shown in Table 2. Quantities of reagents used for PCR amplification are given in Table 4. In case of *CNR1* (rs806368) the PCR products were incubated at 55 °C for 4 h with restriction enzyme BseGI (BtsCI) (Cat # ER0871, ThermoFisher Scientific) which cleaves in the presence of major allele T into fragments of 248 bp and 154 bp size whereas in presence of C allele the 401 bp fragment remain uncut. Then these restriction fragments were visualized on 2% agarose gel in horizontal electrophoresis (Cleaver Scientific, Rugby, UK; catalog number MSMINI10) as shown in Figure 1. In the case of *ACHE*, the incubation of PCR product was done for 16 h at 37 °C in the presence of restriction enzyme Psp5II (PpuMI) (Cat # ER0761, Thermofisher Scientific). Cleavage of Psp5II occur in presence of major allele C which produces fragments of 224 bp and 141 bp size although in presence of T allele the fragment of 365 bp remains uncut. The restriction products were visualized on 2% gel as shown in Figure 1 and Figure 2.

### 2.6. Statistical Analysis

Genotypes and allelic frequencies between addicts and non-addicts were analyzed by Fisher exact test. To assess the association effect in different inheritance models, the Odds ratio with 95% Confidence Interval for both SNPs was calculated. Genotype frequencies were evaluated for deviations from Hardy–Weinberg Equilibrium (HWE) in addicted and non-addicted groups by means of goodness of fit Chi-square test (http://www.had2know.com/academics/hardy-weinberg-equillibriumcalculator-2-alleles.html). GraphPad Prism 5 and online available software (http://vassarstats.net/fisher2x3.html) were utilized for calculations.

## 3. Results

### 3.1. Docking Analysis

**Figure 3:**
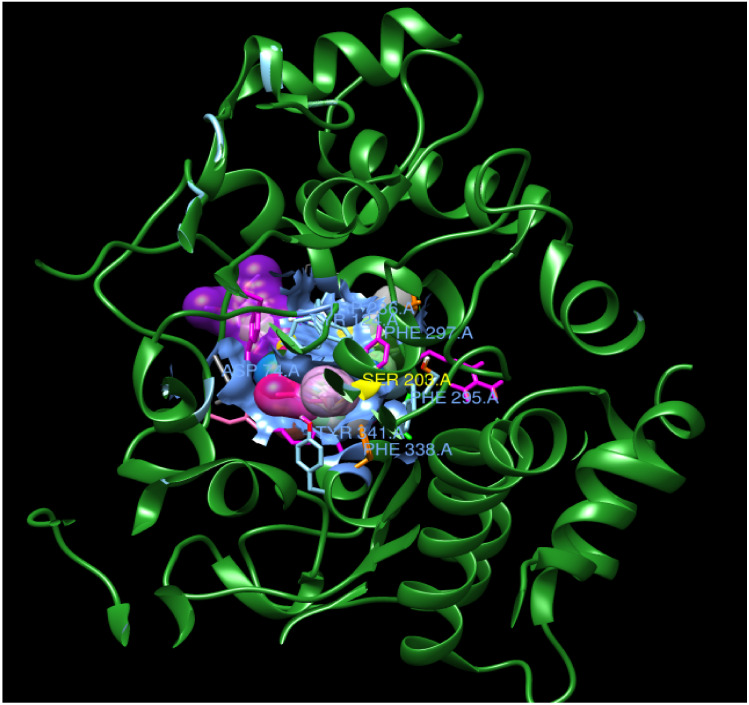
Complex of all ligands with receptor acetylcholinesterase. The receptor is shown in green and all the ligands are depicted in different colors on the same location with the receptor.

**Figure 4:**
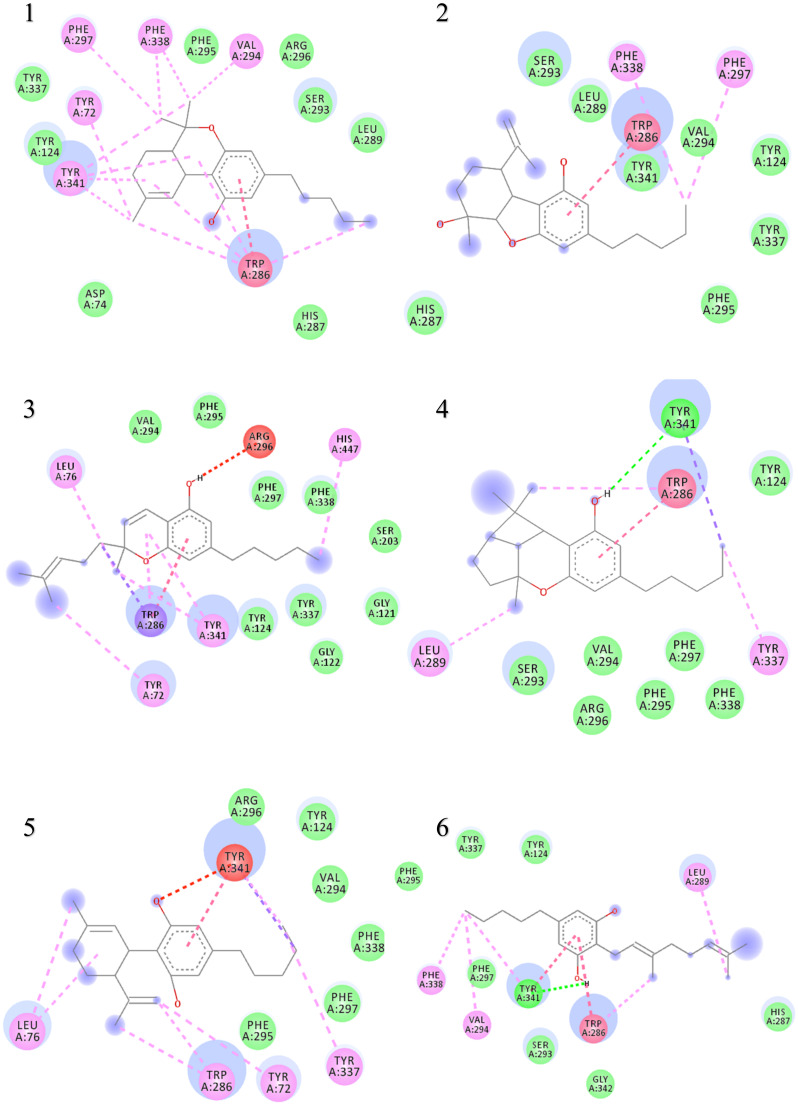

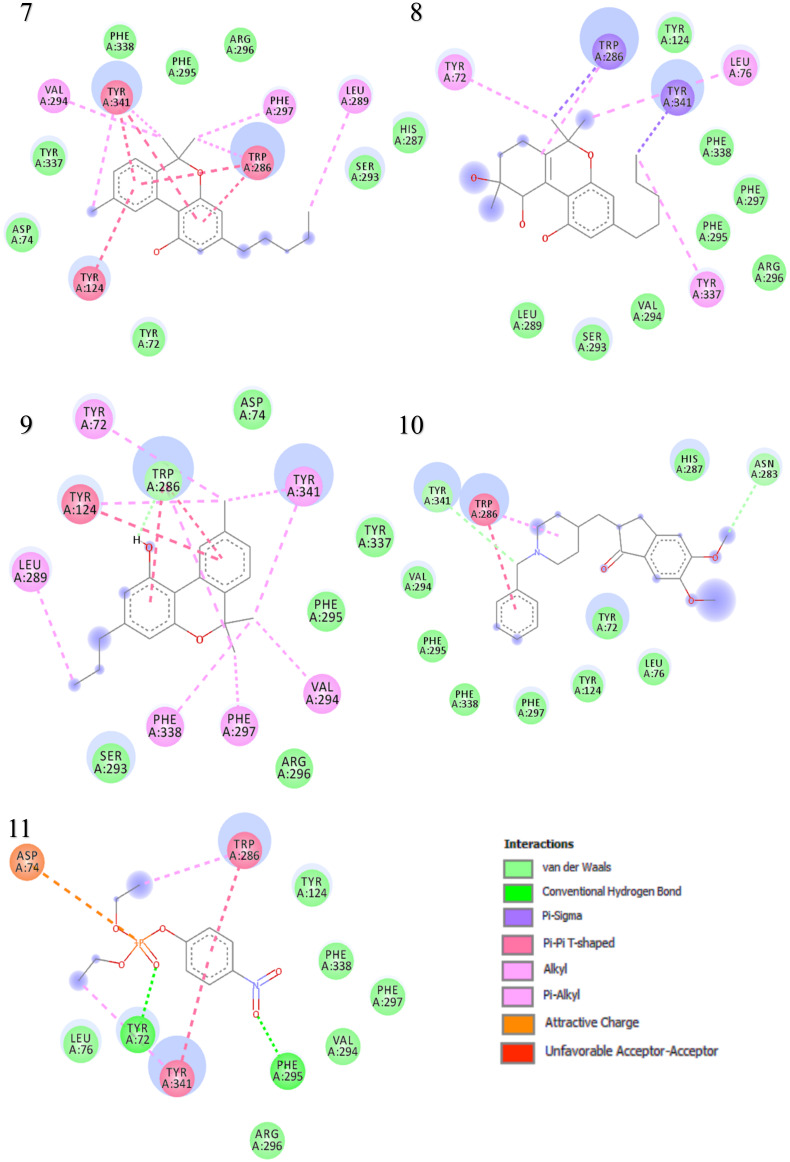
Two-dimensional binding representation of AChE with different ligands: (1) THC (tetrahydrocannabinol), (2) cannabielsoin, (3) Cannabichromene, (4) Cannabicyclol, (5) Cannabidiol, (6) Cannabigerol, (7) Cannabinol, (8) Cannabitriol, (9) Cannabivarin, (10) Donepezil, (11) Paraoxon.

The binding interactions and binding energies for ligands docked against acetylcholinesterase are summarized in Table 5. The free binding energies of donepezil and paraoxon were −8.3 and −6.1 kcal/mol, respectively. Tetrahydrocannabinol (THC) had the lowest binding energy (−9.3 kcal/mol) compared to all other investigated ligands of cannabis. Table 4 lists various kinds of molecular interactions of cannabis ligands with acetylcholinesterase. Figure 3 shows complex which was formed by loading all the ligand, one by one, on receptor using UCSF chimera. It is clearly visible from Figure 3 that all the ligands have taken the same binding pocket as reported residues. 2D binding interactions of all the ligands with acetylcholinesterase receptor are shown in Figure 4.

When the results were compared with the pre-reported interactions of binding residues, it was seen that tyr 341 and phe 297 showing H-bonding between ligand-receptor were present in binding pocket of acetylcholinesterase. Likewise, π-π interactions residue trp 286, tyr124 and 341 were also observed.

### 3.2. LogP Determination

Table 6 shows the logP values that is predicted log of the octanol/water partition coefficient of the understudied biomolecules of cannabis. All of them are highly lipophilic in comparison to donepezil, a drug used for the treatment of Alzheimer.

### 3.3. AChE Activity in Addicted and Non-Addicted Cohort

Status of AChE in addicted and non-addicted individuals is shown in Table 7. AChE enzyme activity in addicted and non-addicted was not different (0.16 μmol/L/min).

### 3.4. Association Analysis of CNR1 rs806368 and ACHE rs17228602

The genotype distribution of *CNR1* rs806368 in controls was according to Hardy–Weinberg Equilibrium (HWE) (χ^2^ = 0.381, *p* = 0.536), while in addicted cases deviation from HWE was observed (χ^2^ = 5.76, *p* = 0.016). Distribution of genotypes in control and cannabis addicted groups for *ACHE* SNP rs17228602 was in concordance with HWE (χ^2^ = 2.480, *p* = 0.115; χ^2^ = 1.146, *p* = 0.284), respectively.

The statistical analysis of genotype and allele frequencies for rs806368 are summarized in Table 8. No significant difference in genotype frequencies was found between cannabis addicts and non-addicts (ϰ^2^ = 2.872, *p* = 0.2379). No homozygote of minor allele C was observed both in cannabis addicts and non-addicts and so no recessive model was evaluated. There was no statistically significant association of rs806368 with risk of cannabis addiction when detected in dominant and allelic models (DM: OR = 0.7403, 95%CI = 0.3072–1.784, *p* = 0.502; Allele OR = 0.9384, 95%CI = 0.4663–1.888, *p* = 0.8585).

Table 9 shows the results of allele and genotype frequencies of *AChE* rs17228602 in cannabis addicts and non-addicts. There was no statistical difference for genotype and allele frequencies of rs17228602 in non-addicts and cannabis addicts (ϰ^2^ = 1.180, *p* = 0.277, Consequently, no significant statistical association was found with addiction vulnerability in any of the inheritance models in cannabis addicts and non-addicts (Table 8). The genotype frequency of CT was lower in addicts than in non-addicts while no TT genotype was observed in both addicts and non-addicts (Table 8). The allele frequency of major allele C was observed to be 86.73% and 81.63% in cannabis addicts and non-addicts, respectively while T minor allele was found to be lower in cannabis addicts as compared to non-addicts (13.27% and 18.37%, respectively).

## 4. Discussion

Acetylcholinesterase has been investigated for association with mental disorders and psychosis since long [36]. AChE is reported to be depressed in depression [37], and some other neurological disorders [38] and is known to interact with many drugs of abuse. It can hydrolyze heroin, a well known substance abuse drug [39], though morphine inhibits AChE [40]. Similarly, the psychoactive effect of cannabis has been attributed to its primary psychoactive constituent, delta-9-tetrahydrocannabinol [19] which is reported as potent AChE inhibitor [41]. Acetylcholinesterase, a hydrolyzing enzyme of the cholinergic neurotransmitter ACh has been a target for cannabis or its constituents since decades [18,42,43,44] but still there is paucity of research in human subjects. Some studies have investigated the cannabis effect on AChE and have reported conflicting results. For instance, Abdel-Salam and Khadrawy [18] reported a decrease in serum AChE activity in rats when subcutaneously treated with cannabis resin. Sadaf et al. and Javed et al. [25,45] reported elevated AChE activity in hashish users. Luthra et al. [46] found that prolong treatment (180 days) in rats caused slight elevation of AChE compared to moderate inhibition by treatment up to 90 days in the brain of male rats. Ghosh et al. [47] reported the surge in AChE in rat brains after acute administration of THC.

Meanwhile, cannabis has been long known for its medicinal applications but uncharacterized physiological interactions of its constituents in the human body and, social stigma associated with substance-abuse has constrained its use in wide-spread clinical settings. In addition, studies mostly been limited to THC or cannabidiol (CBD) constituents of cannabis. Thus, identification and characterization of different bioactive molecules of cannabis is required for its intended application in therapeutics of pathophysiology. Furthermore, genetic heterogeneity has come to be recognized as a source of variable drug response in recent years and may be considered as one of the aetiology of chemical action. In present study, apart from THC and CBD, seven other cannabinoids; Cannabinol (CBN), Cannabicyclol (CBC), Cannabitriol (CBT), Cannabielsoin (CBE), Cannabigerol (CBG), Cannabichromene (CBC), Cannabivarin (CBDV) were evaluated to find their AChE inhibitory capabilities and lipophilicity and compared with donepezil and paraoxon, known AChE inhibitors. AChE activity was measured in human cannabis addicted subjects. In addition, tentative association of cannabis consumption/addiction and cannabinoid receptor 1 gene (*CNR1}* SNP) rs806368 and AChE gene SNP rs17228602 was assessed and found no association. To the best of our knowledge, these are studied for the first time which may open new direction of studies and discussion in future. Our results show that not only THC and CBD are AChE inhibitors rather all other studied cannabinoids have potency to inhibit AChE in this order THC > CBN = CBDV > CBL > CBT > CBE > CBC > CBD > CBG. The compounds rank in following order for lipophilicity; CBG > CBC > CBT > CBD > CBE > THC > CBDV > CBN > CBL. THC has been reported to cause inhibition of AChE by molecular docking previously [41,48]. Some of them (THC, CBN, CBDV, CBL, CBT) were found to be either more or, almost similar potent inhibitors when compared with donepezil; first line drug for treatment of Alzheimer.

Though inhibitory action of AChE was not observed in vivo as reported by some earlier studies and in contrast to in silico molecular docking here in this work. This apparent disparity between in silico prediction and in vivo finding might be due to antagonizing behavior of other components of cannabis like cannabidiol [20]. This inhibitory, stimulatory or no effect may be based on the dose and duration of cannabis exposure in addition to the quantity of bioactive constituents in cannabis. Moreover, various interactions among different neurotransmitters in the body could also contribute to that. For instance, THC if acted through CB1 receptors, would cause the release of dopamine [49]. Increase in dopamine may decrease the AChE or increase the acetylcholine [50]. Apart from these interactions of various neurotransmitters, SNP polymorphisms in genes may also complicate differential physiology. However, no polymorphism in the studied group was found for chosen ACHE and CNR1 SNPs, though patterns of allelic shift were noted in rs806378 of CNR1 gene. The overall findings suggest that cholinergic and cannabinoid interaction is very complex and cannot be interpreted with cholinergic enzymes only. From the literature it is evident that some interactions do exists [18,19,50]. Though previous conventionally research has focused on two major components of cannabis i.e., THC and CBD as potential candidate for therapeutic applications but in silico prediction could unravel many others. However, in silico prediction could not be perhaps translated to in vivo human physiology because of various antagonist constituents of the cannabis. Not only the antagonistic attributes natural physiological conditions, and pharmacokinetics of the molecules like absorption, distribution, metabolic fate and elimination also significantly influence the efficacy which may vary in different experimental models. Therefore, in silico and in vitro findings even for an individual component of cannabis should be cautiously translated for human applications. Future work with participants from multiple ethnic backgrounds and with well-documented drug-usage patterns need to be carried out for more tangible understanding. Despite certain limitations, the study will open the discussion and further investigation for the possible therapeutic application of AChE inhibitors molecules of cannabis other than THC and cannabidiol. For instance, if we look at the data, CBT has better AChE inhibitory potency than CBD and more lipophilic than THC and CBD, but no physiological study could be traced for CBT. The literature search of cannabitriol on PubMed retrieved only four papers related with isolation and structure only.

## 5. Conclusions

Cannabis addiction was not found to alter the acetylcholinesterase in the blood of cannabis users, though in silico molecular binding of its constituents predicted the inhibitory actions. This apparent predicted and observed discrepancy in AChE could be a masking effect of different biomolecules when present together. Association analysis did not show cannabis addiction vulnerability with SNPs rs806368 and rs17228602. However, variation trends in the allelic frequencies were found in the allelic model of rs806368 with possible pharmacogenetics potential and need more exploration in this regard. Further investigation on CBT for AChE inhibitor based therapeutic application for neuronal disorder is suggested. A more robust conclusion can be drawn by further studies with increased number of addicted and non-addicted individuals and different combination of cannabis biomolecules with predicted potential to inhibit AChE.

## Figures and Tables

**Table 1:** Binding pockets of selected inhibited acetylcholinesterase (AChE) protein.

	Catalytic Triad	Acyl Pocket	Peripheral Anionic Site	Phosphyl Site
Acetylcholinesterase (4PQE)	Ser-203 Glu-334 His-447	Phe297 Phe338 Phe295 Trp286	Ser125 Tyr341 Asp74 Tyr124 Trp286 Tyr237 Glu285	Val294 Ser293 Trp286 Tyr72 Ser294 Tyr337

**Table 2:** Parameters used in Vina search space for docking.

Center (Å)		Dimensions (Å)	
X	–27.5501	X	65.5753
Y	–0.2162	Y	62.4444
Z	50.3675	Z	57.9704

**Table 3:** Primer sequences for single nucleotide polymorphisms (SNPs) rs806368 and rs17228602.

Primer’s ID	Primer’s Sequences	Tm	Product Size
**rs806368**			
*CNR1*-SNP1 F	5′GCAACGATGTTACCAGCTCAAC3′	62.0 C	
*CNR1*-SNP1 R	5′ATTGTCTTGCACTGGCCTTCTG3′	63.8 C	401 bp
**rs17228602**			
*ACHE*-SNP2 F	5′GAGGAGGAGAAAAGAATGACC3′	56.9 C	
*ACHE*-SNP2 R	5′TCCTCTAATGAGTGGTCGGAC3′	59.2 C	365 bp

**Table 4:** Quantity of reagents and procedure used for the genotyping of rs806368 and rs17228602.

Reagents (Total Volume = 25 µL)	Quantity of Reagents for rs806368 (µL)	Quantity of Reagents for rs17228602 (µL)
Taq Buffer	2.5 (1 X)	2.5 (1 X)
MgCl_2_	3.0 (1.5 mM)	3.0 (1.5 mM)
dNTPs	0.5(2.5 mM)	0.5 (2.5 mM)
Primers	F = 0.5 (10 pmol) R = 0.5 (10 pmol)	F = 0.5 (10 pmol) R = 0.5 (10 pmol)
PCR water	14.5	14.5
DNA sample	3.0 (25 ng/uL)	3.0 (30 ng/uL)
Taq polymerase	0.5 (5 units)	0.5 (5 units)
Thermal Profile		
Denaturation	95 °C for 5 min 95 °C for 30 sec	95 °C for 5 min 95 °C for 30 sec
Annealing	57 °C for 30 sec	56.5 °C for 30 sec
Extension	72 °C for 1 min 72 °C for 7 min	72 °C for 45 sec 72 °C for 7 min
Total cycles	35 X	35 X

**Table 5:** Binding energies of Cannabis components in blind docking against AChE.

Ligand Name	Free Binding Energy (Kcal/mol)	Hydrogen Bonding		π-π Interactions	Van der Waals Interactions
		Interacting Residues	Bond Distance (Ǻ)		
THC	−9.3	No H bond	N/A	Trp286	Tyr337, Tyr124, Asp74, His 287, Leu289, Ser293, Arg296, Phe295
Cannabielsoin	−7.7	No H bond	N/A	Trp286	Ser293, Leu289, His287, Phe295, Tyr337, Tyr124, Tyr341, Val294
Cannabichromene	−7.6	No H bond	N/A	N/A	Val294, Phe295, Phe297, Phe338, Ser203, Gly121, Tyr124, Tyr337, Gly122
Cannabicyclol	−8.1	TYR 341	3.51	Trp286	Tyr124, Ser293, Phe297, Phe338, Phe295, Val294, Arg296
Cannabidiol	−7.5	No H bond	N/A	N/A	Arg296, Tyr124, Val294, Phe338, Phe297, Phe295
Cannabigerol	−7.0	TYR 341	4.73	Trp286	Phe295, Tyr124, Tyr337, Phe297, Ser293, Gly342, His287
Cannabinol	−8.6	No H bond	N/A	Tyr341, Tyr124, Trp286	Arg296, Phe295, Phe338, Tyr337, Asp74, Tyr72, Ser293
Cannabitriol	−7.9	No H bond	N/A	N/A	His287, Tyr124, Phe338, Phe297, Phe295, Arg296, Val294, Ser293, Leu289
Cannabivarin	−8.6	No H bond	N/A	Tyr124	Asp74, Tyr337, Phe295, Arg296, Ser293
Donepezil	−8.3	No H bond	N/A	Trp286	His287, Tyr72, Leu76, Tyr124, Phe297, Phe338, Phe295, Val 294
Paraoxon	−6.1	TYR72 PHE 295	5.58 5.36	Trp286 Tyr341	Leu76, Arg296, Val294, Phe297, Phe338, Tyr124

**Table 6:** LogP values and molecular structures of constituents of cannabis, donepezil and paraoxon.

Chemical Name	logP	Chemical Structure
Δ9-Tetrahydrocannabinol (THC) Mol. Weight = 314.5 g/mol	7.26	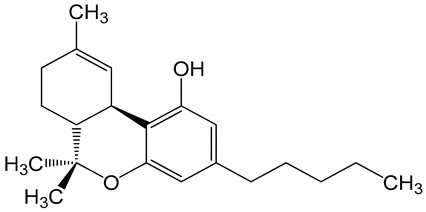
Cannabinol (CBN) Mol. Weight = 310.4 g/mol	5.58	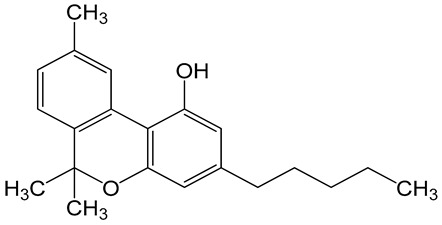
Cannabidiol (CBD) Mol. Weight = 314.5 g/mol	7.75	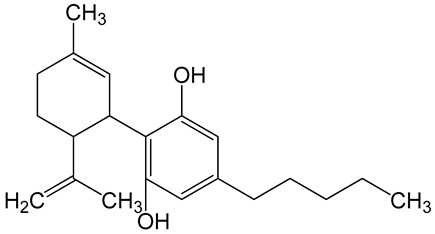
Cannabicyclol (CBL) Mol. Weight = 314.5 g/mol	4.96	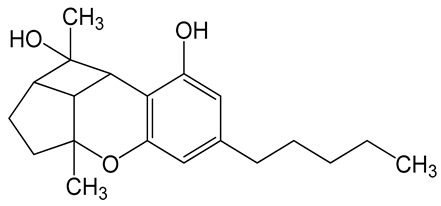
Cannabitriol (CBT) Mol. Weight = 346.5 g/mol	8.04	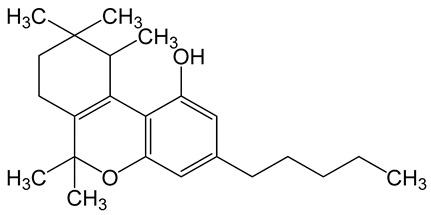
Cannabielsoin (CBE) Mol. Weight = 330.5 g/mol	7.64	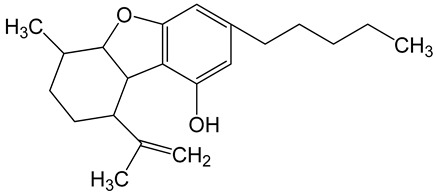
Cannabigerol (CBG) Mol. Weight = 316.5 g/mol	8.59	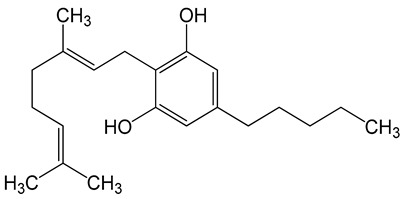
Cannabichromene (CBC) Mol. Weight = 314.5 g/mol	8.28	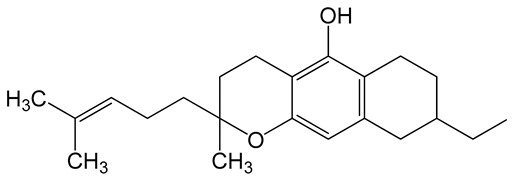
Cannabivarin (CBDV) Mol. Weight = 282.4 g/mol	6.98	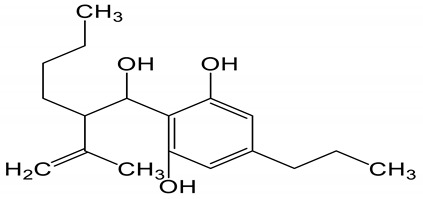
Paraoxon Mol. Weight = 275.19 g/mol	2.07	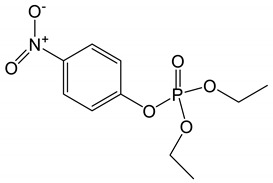
Donepezil Mol. Weight = 379.5 g/mol	3.39	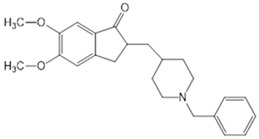

**Table 7:** Acetylcholinesterase activity in non-addicted and cannabis addicted subjects.

Groups	Mean (µmol/L/min)	SD	SE	95% Confidence Interval for Mean	
				Lower Bound	Upper Bound
Non-addicted	0.016 (n = 31)	0.011	0.002	0.011	0.020
Cannabis addicted	0.016 (n = 41)	0.003	0.001	0.014	0.017

**Table 8:** Association analysis of *CNR1* rs806368 between cannabis addicts and non-addicts.

Genotype	Cannabis Addicts N = 40 (%)	Non-Addicts n = 40 (%)	Odd Ratio (95% CI) *p*-Value	*χ^2^* (*p*-Value) Significant ≥ 0.05
TT	18 (45)	21 (52.5)		2.872 (0.2379)
TC	22 (55)	17 (42.5)		
CC	0 (0%)	02 (5)		
Dominant model	18/22	21/19	0.740 (0.307–1.784) (0.502)	0.450 (0.502)
Allele				
T	58/80 (72.50)	59/80 (73.75)	0.938 (0.466–1.888) (0.858)	0.0318 (0.8585)
C	22/80 (27.50)	21/80 (26.25)		

**Table 9:** Association analysis of ACHE rs17228602 between cannabis addicts and non-addicts.

	Cannabis Addicts *n = 49 (*%)	Non-Addicts *N = 49(*%)	Odd Ratio (95% CI) *p*-Value	*χ^2^* (*p*-Value) Significant ≥ 0.05
CC	36 (73.47)	31 (63.27)		1.180 (0.277)
CT	13 (26.53)	18 (36.73)		
TT	0 (0%)	0 (0%)		
Dominant model	36/13	31/18	0.623 (0.263–1.470) (0.277)	1.180 (0.277)
Allele				
C	85/98 (86.73)	80/98 (81.63)	1.471 (0.677–3.197) 0.327	0.958 (0.328)
T	13/98 (13.27)	18/98 (18.37)		
	Cannabis Addicts *n = 49 (*%)	Non-Addicts *N = 49(*%)	Odd ratio (95% CI) *p*-value	*χ^2^* (*p*-value) significant ≥ 0.05
CC	36 (73.47)	31 (63.27)		1.180 (0.277)
CT	13 (26.53)	18 (36.73)		
TT	0 (0%)	0 (0%)		
Dominant model	36/13	31/18	0.623 (0.263–1.470) (0.277)	1.180 (0.277)
Allele				
C	85/98 (86.73)	80/98 (81.63)	1.471 (0.677–3.197) 0.327	0.958 (0.328)
T	13/98 (13.27)	18/98 (18.37)		

## References

[1] ElSohly M.A., Radwan M.M., Gul W., Chandra S., Galal A. (2017). Phytochemistry of *Cannabis sativa* L.. Prog. Chem. Org. Nat. Prod..

[2] Burggren A.C., Shirazi A., Ginder N., London E.D. (2019). Cannabis effects on brain structure, function, and cognition: Considerations for medical uses of cannabis and its derivatives. Am. J. Drug Alcohol Abus..

[3] Di Marzo V. (2018). New approaches and challenges to targeting the endocannabinoid system. Nat. Rev. Drug Discov..

[4] Kokkevi A., Gabhainn S.N., Spyropoulou M. (2006). Early Initiation of Cannabis Use: A Cross-national European Perspective. J. Adolesc. Health.

[5] Russo E.B., Marcu J. (2017). Cannabis Pharmacology: The Usual Suspects and a Few Promising Leads. Adv. Pharmacol..

[6] Maroon J., Bost J. (2018). Review of the neurological benefits of phytocannabinoids. Surg. Neurol. Int..

[7] Pertwee R.G. (2005). The therapeutic potential of drugs that target cannabinoid receptors or modulate the tissue levels or actions of endocannabinoids. AAPS J..

[8] Vallée A., Lecarpentier Y., Guillevin R., Vallée J.-N. (2017). Effects of cannabidiol interactions with Wnt/β-catenin pathway and PPARγ on oxidative stress and neuroinflammation in Alzheimer’s disease. Acta Biochim. Biophys. Sin..

[9] Watt G., Karl T. (2017). In vivo Evidence for Therapeutic Properties of Cannabidiol (CBD) for Alzheimer’s Disease. Front. Pharmacol..

[10] Calabrese E.J., Rubio-Casillas A. (2018). Biphasic effects of THC in memory and cognition. Eur. J. Clin. Invest..

[11] Green B., Kavanagh D., Young R. (2003). Being stoned: A review of self-reported cannabis effects. Drug Alcohol Rev..

[12] Mineur Y.S., Obayemi A., Wigestrand M.B., Fote G.M., Calarco C.A., Li A.M., Picciotto M.R. (2013). Cholinergic signaling in the hippocampus regulates social stress resilience and anxiety- and depression-like behavior. Proc. Natl. Acad. Sci. USA.

[13] Brady K.T., Gray K.M., Tolliver B.K. (2011). Cognitive enhancers in the treatment of substance use disorders: Clinical evidence. Pharmacol. Biochem. Behav..

[14] Terranova J.-P., Storme J.-J., Lafon N., Pério A., Rinaldi-Carmona M., Le Fur G., Soubrié P. (1996). Improvement of memory in rodents by the selective CB1 cannabinoid receptor antagonist, SR 141716. Psychopharmacology.

[15] Revuelta A.V., Cheney D.L., Costa E., Lander N., Mechoulam R. (1980). Reduction of hippocampal acetylcholine turnover in rats treated with (−)-delta 8-tetrahydrocannabinol and its 1′,2′-dimethyl-heptyl homolog. Brain Res..

[16] Pacher P., Bátkai S., Kunos G. (2006). The endocannabinoid system as an emerging target of pharmacotherapy. Pharmacol. Rev..

[17] Coutselinis A., Michalodimitrakis M. (1981). Acetylcholinesterase Activity after Hashish Smoking. Clin. Toxicol..

[18] Abdel-Salam O.M.E., Youness E.R., Khadrawy Y.A., Sleem A.A. (2016). Acetylcholinesterase, butyrylcholinesterase and paraoxonase 1 activities in rats treated with cannabis, tramadol or both. Asian Pac. J. Trop. Med..

[19] Moss D.E., Peck P.L., Salome R. (1978). Tetrahydrocannabinol and acetylcholinesterase. Pharmacol. Biochem. Behav..

[20] Kim S.H., Yang J.W., Kim K.H., Kim J.U., Yook T.H. (2019). A Review on Studies of Marijuana for Alzheimer’s Disease – Focusing on CBD, THC. J. Pharmacopunct..

[21] Corley R.P., Zeiger J.S., Crowley T., Ehringer M.A., Hewitt J.K., Hopfer C.J., Lessem J., McQueen M.B., Rhee S.H., Smolen A. (2008). Association of candidate genes with antisocial drug dependence in adolescents. Drug Alcohol Depend..

[22] Zuo L., Kranzler H.R., Luo X., Covault J., Gelernter J. (2007). CNR1 Variation Modulates Risk for Drug and Alcohol Dependence. Biol. Psychiatry.

[23] Peiró A.M., García-Gutiérrez M.S., Planelles B., Femenía T., Mingote C., Jiménez-Treviño L., Martínez-Barrondo S., García-Portilla M.P., Saiz P.A., Bobes J. (2020). Association of cannabinoid receptor genes (*CNR1* and *CNR2*) polymorphisms and panic disorder. Anxiety Stress Coping.

[24] Chen X., Williamson V.S., An S.-S., Hettema J.M., Aggen S.H., Neale M.C., Kendler K.S. (2008). Cannabinoid Receptor 1 Gene Association with Nicotine Dependence. Arch. Gen. Psychiatry.

[25] Javed T., Habib R., Ghafoor S., Rumman B., Awan S., Ntepe L.J.M., Batool S., Nurulain S.M. (2019). Association of status of acetylcholinesterase and ACHE gene 3′ UTR variants (rs17228602, rs17228616) with drug addiction vulnerability in pakistani population. Chem. Biol. Interact..

[26] Waqar M., Batool S. (2015). In silico analysis of binding of neurotoxic venom ligands with acetylcholinesterase for therapeutic use in treatment of Alzheimer’s disease. J. Theor. Biol..

[27] Berman H.M., Westbrook J., Feng Z., Gilliland G., Bhat T.N., Weissig H., Shindyalov I.N., Bourne P.E. (2000). The Protein Data Bank. Nucleic Acids Res..

[28] Pettersen E.F., Goddard T.D., Huang C.C., Couch G.S., Greenblatt D.M., Meng E.C., Ferrin T.E. (2004). UCSF Chimera—A visualization system for exploratory research and analysis. J. Comput. Chem..

[29] Khazanov N.A., Carlson H.A. (2013). Exploring the Composition of Protein-Ligand Binding Sites on a Large Scale. PLoS Comput. Biol..

[30] Kim S., Chen J., Cheng T., Gindulyte A., He J., He S., Li Q., Shoemaker B.A., Thiessen P.A., Yu B. (2019). PubChem 2019 update: Improved access to chemical data. Nucleic Acids Res..

[31] Pence H.E., Williams A. (2010). ChemSpider: An Online Chemical Information Resource. J. Chem. Educ..

[32] Dallakyan S., Olson A.J., Hempel J.E., Williams C.H., Hong C.C. (2015). Small-Molecule Library Screening by Docking with PyRx. Chemical Biology: Methods and Protocols.

[33] Darvas F., Marokházti S., Kormos P., Gururaj K., Kalász H., Papp Á., Erhardt P.W. (1999). MetabolExpert: Its Use in Metabolism Research and in Combinatorial Chemistry. Drug Metabolism.

[34] Worek F., Mast U., Kiderlen D., Diepold C., Eyer P. (1999). Improved determination of acetylcholinesterase activity in human whole blood. Clin. Chim. Acta Int. J. Clin. Chem..

[35] Lahiri D.K., Nurnberger J.I. (1991). A rapid non-enzymatic method for the preparation of HMW DNA from blood for RFLP studies. Nucleic Acids Res..

[36] Wright C.I., Sabine J.C. (1943). The Inactivation of Cholinesterase by Morphine, Dilaudid, Codeine and Desomorphine. J. Pharmacol. Exp. Ther..

[37] Suarez-Lopez J.R., Hood N., Suárez-Torres J., Gahagan S., Gunnar M.R., López-Paredes D. (2019). Associations of acetylcholinesterase activity with depression and anxiety symptoms among adolescents growing up near pesticide spray sites. Int. J. Hyg. Environ. Health.

[38] Fritze J., Beckmann H. (1987). Erythrocyte acetylcholinesterase in psychiatric disorders and controls. Biol. Psychiatry.

[39] Kim K., Yao J., Jin Z., Zheng F., Zhan C.-G. (2018). Kinetic characterization of cholinesterases and a therapeutically valuable cocaine hydrolase for their catalytic activities against heroin and its metabolite 6-monoacetylmorphine. Chem. Biol. Interact..

[40] Sim M.K., Chua M.E. (1986). Inhibition of acetylcholinesterase by various opioids. Clin. Exp. Pharmacol. Physiol..

[41] Eubanks L.M., Rogers C.J., Beuscher A.E., Koob G.F., Olson A.J., Dickerson T.J., Janda K.D. (2006). A Molecular Link between the Active Component of Marijuana and Alzheimer’s Disease Pathology. Mol. Pharm..

[42] Yoshimura H., Fujiwara M., Ueki S. (1974). Biochemical correlates in mouse-killing behavior of the rat: brain acetylcholine and acetylcholinesterase after administration of A 9-tetrahydrocannabinol. Brain Res..

[43] Nestler E.J. (2013). Cellular basis of memory for addiction. Dialogues Clin. Neurosci..

[44] Brown A.N., Feng J. (2017). Drug Addiction and DNA Modifications. Adv. Exp. Med. Biol..

[45] Munir S., Habib R., Awan S., Bibi N., Tanveer A., Batool S., Nurulain S.M. (2019). Biochemical Analysis and Association of Butyrylcholinesterase SNPs rs3495 and rs1803274 with Substance Abuse Disorder. J. Mol. Neurosci..

[46] Luthra U.L., Rosenkrantz H., Heyman I.A., Braude M.C. (1975). Differential neurochemistry and temporal pattern in rats treated orally with delta9-tetrahydrocannabinol for periods up to six months. Toxicol. Appl. Pharmacol..

[47] Ghosh J.J., Poddar M.K., Nag D., Biswas B. (1975). Delta-9-tetrahydrocannabinol action and neuronal membrane-bound enzymes. Prog. Brain Res..

[48] Seniya C., Khan G.J., Uchadia K. (2014). Identification of potential herbal inhibitor of acetylcholinesterase associated Alzheimer’s disorders using molecular docking and molecular dynamics simulation. Biochem. Res. Int..

[49] Bossong M.G., Mehta M.A., van Berckel B.N.M., Howes O.D., Kahn R.S., Stokes P.R.A. (2015). Further human evidence for striatal dopamine release induced by administration of Δ9-tetrahydrocannabinol (THC): Selectivity to limbic striatum. Psychopharmacology.

[50] De Boer A.G., Wijker W., Speelman J.D., de Haes J.C. (1996). Quality of life in patients with Parkinson’s disease: Development of a questionnaire. J. Neurol. Neurosurg. Psychiatry.

